# Genetic prediction of complex traits: integrating infinitesimal and marked genetic effects

**DOI:** 10.1007/s10709-013-9722-9

**Published:** 2013-05-30

**Authors:** Clément Carré, Fabrice Gamboa, David Cros, John Michael Hickey, Gregor Gorjanc, Eduardo Manfredi

**Affiliations:** 1 UR 631 SAGA, INRA Toulouse, B.P. 52627, Auzeville, 31326 Castanet-Tolosan Cedex, France; 2 IMT, Université Paul Sabatier, Toulouse, France; 3 AGAP, CIRAD, Montpellier, France; 4 Biometrics and Statistics Unit, International Maize and Wheat Improvement Center (CIMMYT), 06600 Mexico, D.F. Mexico; 5 Animal Science Department, Biotechnical Faculty, University of Ljubljana, Ljubljana, Slovenia

**Keywords:** Genetic prediction, Genomic selection, SNP, Mendelian sampling

## Abstract

Genetic prediction for complex traits is usually based on models including individual (infinitesimal) or marker effects. Here, we concentrate on models including both the individual and the marker effects. In particular, we develop a “Mendelian segregation” model combining infinitesimal effects for base individuals and realized Mendelian sampling in descendants described by the available DNA data. The model is illustrated with an example and the analyses of a public simulated data file. Further, the potential contribution of such models is assessed by simulation. Accuracy, measured as the correlation between true (simulated) and predicted genetic values, was similar for all models compared under different genetic backgrounds. As expected, the segregation model is worthwhile when markers capture a low fraction of total genetic variance.

## Introduction

In recent years, new knowledge on molecular genetics and the rapid evolution of sequencing and genotyping technology has renewed the interest on genetic prediction of complex traits. It should be recalled, however, that genetic prediction of complex traits has been a traditional field in animal and plant breeding since the 40’s in the framework of the Selection Index (SI) theory (e.g., Hazel 1943), extended later to the “best linear unbiased prediction” (BLUP; Henderson 1975). These genetic prediction methods, without DNA data, were based on the “individual” model where covariances amongst phenotypes of related individuals are translated into unobserved covariances amongst genetic values, via theoretical relatedness coefficients amongst individuals. Anticipating the availability of low-cost whole genome DNA data, Meuwissen et al. (2001) proposed “marker” models where many markers’ genotypes represent genetic effects, while the individuals are not explicitly specified in the model. We concentrate here on a third group of models including both “marker” and “individual” effects. We first recall the families of models proposed for genetic prediction and then we develop a novel model, which is illustrated with an example. Then, we assess the relative performance of the novel model in relation to the marker model for different genetic scenarios, and we report results of the analyses of a public simulated sample. Finally, originality, limits and possible extensions of the model are discussed.

## Individual models for genetic prediction

Both SI and BLUP are applied to the “infinitesimal” (or polygenic) genetic model which in its simplest version is “phenotype = mean + additive genetic value + residual”. This model has been called “polygenic” or “infinitesimal” since the additive genetic value is the sum of the effects, assumed to be small and homogeneous, of numerous genes on the phenotype. In the statistical model, built from the genetic model, “individual effects” are used to represent additive genetic effects, and they are assumed random because genotype configurations of individuals arise through random processes:

1 $${\mathbf{y}} = {\varvec{\mu}} + {\mathbf{Zu}} +  {\mathbf{e}} $$

***y*** is a vector of phenotypes

***μ*** is a constant vector (assumed known in SI and estimated in BLUP)

***Z*** is an incidence matrix of order $N_{y} \left( {\text{phenotypes}} \right) \times N_{i} \left( {\text{individuals}} \right),$ relating each of the $N_{y}$ phenotypes to each of the measured individuals. For simplicity, we assume only one measure per individual. In standard BLUP technology $${\mathbf{Z}} = \left[ {\begin{array}{*{20}c} {\mathbf{0}} & {\mathbf{I}} & {\mathbf{0}} \\ \end{array} } \right]$$, i.e., null columns for base individuals without phenotypes, the identity matrix for individuals with phenotypes (when there is a single measure for each individual), and null columns for descendants without phenotype, the usual target of prediction. In this context of genetic prediction, base individuals are defined for a given genealogy as the most distant known ancestors of individuals with recorded phenotypes, i.e., they do not have phenotypes and their parents are unknown.

**u** is a vector of additive genetic effects, with ${\text{Var}}\left( {\mathbf{u}} \right) = {\mathbf{A}}{{\upsigma}}_{\text{u}}^{2}$, with **A** being the relationship matrix amongst individuals.

**e** is a vector of residuals, with ${\text{Var}}\left( {\mathbf{e}} \right) = {\mathbf{I}}{{\upsigma}}_{\text{e}}^{2}$, with **I** being an identity matrix

A further usual assumption is ${\text{Cov}}({\mathbf{e}},{\mathbf{u}}) = 0$.

The only information available to distinguish genetic effects from residuals are the structures of the (co)variance matrices of **u** and **e**. In other words, the model describes a network of phenotypic covariances (observed) which are translated into genetic covariances (unobserved) via the theoretical genetic model, in particular the relatedness coefficients in the relationship matrix **A**.

## Marker and individual models

With molecular data available, prediction models evolved to include this new information (e.g., Fernando and Grossman 1989; Meuwissen et al. 2001). Fernando and Grossman (1989) proposed a prediction model which included several genetic effects: an infinitesimal effect **u** plus haplotype effects of maternal and paternal origin at marked quantitative trait loci (QTL) positions. Their model was reasonably conservative, given the genomic tools available by that time (say, 500 microsatellites to cover the entire genome in farm animals). In this context, they assumed that a marker allele may mark different QTL alleles in different families. Later, with many more markers (10,000 multi-allelic markers), Meuwissen et al. (2001) switched from the previous conservative model to “marker” models exploiting linkage disequilibrium at the population level:

2 $${\mathbf{y}} = {\varvec{\mu}} + {\mathbf{ZWm}} + {\mathbf{e}} $$

where:

**m** is a vector of marked genetic effects (usually termed “marker effects”, although the usual hypothesis is that markers do not have a true effect *per se* on the phenotype)

**W** is a matrix of marker genotypes of order ${\text{N}}_{\text{i }} \left( {\text{individuals}} \right) \times {\text{N}}_{\text{m}} \left( {\text{markers}} \right).$ With biallelic markers such as SNP, usual elements of ***W*** are 0, 1 or 2, the number of, say, the allele “1” of the marker genotype.

Usually assumed (co)variances are:

$$\begin{gathered} {\text{Var}}\left( {\mathbf{m}} \right) = {\mathbf{I}}_{{{\text{N}}_{\text{m}} }} {{\upsigma}}_{\text{m}}^{2} \hfill \\ {\text{Cov}}({\mathbf{e}},{\mathbf{m}}) = {\mathbf{0}} \hfill \\ \end{gathered}$$

where ${\mathbf{I}}_{{{\text{N}}_{\text{m}} }}$ is an identity matrix of order ${\text{N}}_{\text{m}}$.

If we further assume that ${\mathbf{u}} = {\mathbf{Wm}}$ and ${\text{Var}}\left( {\mathbf{u}} \right) = {\mathbf{WW}}^{ '} {{\upsigma}}_{\text{m}}^{2}$, it is possible to compute predictions for **u** with the individual model (1), amended such that the relationship matrix **A** is replaced by the realized “genomic relationship” matrix ${\mathbf{G}} = {\mathbf{WW}}^{ '}$ (VanRaden 2008; Goddard 2009). Application of BLUP to this model has been termed “genomic BLUP” and improvements have been proposed to make assumptions more realistic (departures from the homogeneous variances for marked effects in model (2)) and practical implementations when only part of the individuals are genotyped making necessary to mix the **A** and the **G** matrices for the combined analyses of individuals with or without genotypes (e.g. Aguilar et al. 2011).

## Marker plus individual model

Alternative assumptions in an outbred population are ${\mathbf{u}} \ne {\mathbf{Wm}}$ and ${\text{Var}}\left( {\mathbf{u}} \right) \ne {\mathbf{WW}}^{ '} {{\upsigma}}_{\text{m}}^{2}$. There are theoretical reasons and experimental results to support this point of view. Theoretically, in a Bayesian context, Gianola et al. (2009) claimed that the functional relationship between $\sigma_{u}^{2}$ and $\sigma_{m}^{2}$ is elusive. They did propose simple approximations under Hardy–Weinberg and linkage equilibria (LE) to relate the marked genetic variance and the additive genetic variance as ${{\upsigma}}_{\text{u}}^{2} = 2\mathop \sum \nolimits_{{{\text{i}} = 1}}^{{{\text{N}}_{\text{m}} }} {\text{p}}_{\text{i}} {\text{q}}_{\text{i}} {{\upsigma}}_{\text{m}}^{2}$, where ${\text{p}}_{\text{i}} \;{\text{and}}\; {\text{q}}_{\text{i}}$ are the allelic frequencies for marker i. However, assuming LE is not compatible with the essential assumption of linkage disequilibrium in the context of genome-wide analysis. Furthermore, in most experimental studies, the sum of variances due to marker associations does not add up to the additive genetic variance due to individual infinitesimal effects raising the problem of the “hidden heritability” (e.g., Yang et al. 2011).

The unknown vector **m** represents the effects of unobserved genes that should be marked by observed markers. This model should fit all genome-wide additive effects simultaneously. However, it is not warranted that all the actual additive genetic effects in the studied genome will be effectively traced by the available markers (Yang et al. 2011). Potential problems are poor marker coverage (low density but also insufficient representation of independent DNA segments), rare alleles, small (infinitesimal) gene effects, multi-allelic genes having additive effects that are poorly traced by bi-allelic markers, or other molecular genetics mechanisms. The main assumption is that each marker allele or haplotype is associated with each unobserved QTL allele in identical way for each individual in the studied population. This may be true in some cases but it is not true in general. While an association between a marker and the QTL may be stable within parents and progeny, open populations over several generations are built up by subpopulations, each one with its own QTL allele-marker allele association. Reintroduction of infinitesimal effects in the prediction model is one of the recommended ways to control partially the lack of perfect association between marker alleles and causative alleles (Goddard and Hayes 2009). The model becomes:

3 $${\mathbf{y}} = {\varvec{\mu}} + {\mathbf{Zu}} + {\mathbf{ZWm}} + {\mathbf{e}} $$

with additional assumptions:

$${\text{Var}}\left( {\mathbf{u}} \right) = {\mathbf{R}}{{\upsigma}}_{\text{u}}^{2} ,\;{\text{and}}\;{\text{Cov}}({\mathbf{e}},{\mathbf{u}}) = {\text{Cov}}({\mathbf{u}},{\mathbf{m}}) = {\mathbf{0}},$$

where ${\mathbf{R}}\sigma_{u}^{2}$ is the symmetric (co)-variance matrix of individual effects of order ${\text{N}}_{\text{i}}$. Usually, as in model (1), **R** = **A**, the additive relationship matrix computed theoretically from genealogy data. Note that the terms in model (3) are redundant if it is assumed that **u** = **Wm**.

The idea in model (3) is to include residual genetic values not taken into account by the marked effects **m.** In applications, this model gave better predictions than the marker model (2) (e.g., De los Campos et al. 2009; Duchemin et al. 2012).

## Mendelian segregation model

Here, we develop a model where the genetic value of an individual is a function of infinitesimal effects of ancestors (individuals in the base, with unknown parents) and Mendelian sampling which can be traced by DNA data. In the following it is assumed that all individuals have complete genotype data and all descendants have known parents. We then discuss the departures from this complete data situation.

The model starts as in (3):

$${\mathbf{y}} = {\varvec{\mu}} + {\mathbf{Zu}} + {\mathbf{ZWm}} + {\mathbf{e}}$$

It is convenient to separate individuals in two groups: the base ancestors with unknown parents (indexed by b) and the descendants (indexed by d). We can now expand and decompose the vector of infinitesimal values **u** as:

$${\mathbf{u}} = \left[ {\begin{array}{*{20}c} {{\mathbf{u}}_{\text{b}} } \\ {{\mathbf{u}}_{\text{d}} } \\ \end{array} } \right]$$

Let **P** be a N_i_ × N_i_ matrix with two 1’s in each row, indicating the parents of each individual (rows of **P** for base individuals are null).

We define the matrix **M** as:

$${\mathbf{M}} = \left( {{\mathbf{I}} - \frac{1}{2}{\mathbf{P}}} \right)$$

The matrix **M** is interpretable in biology (each row of **M** represents the individual minus half the sum of parents) and in mathematics since **M** has the form of a Laplacian matrix, representing the pedigree graph, with **P** being the adjacency matrix with elements equal to 1 at the intersection of adjacent nodes (parent and progeny nodes) or 0 otherwise.

**Fig. 1:**
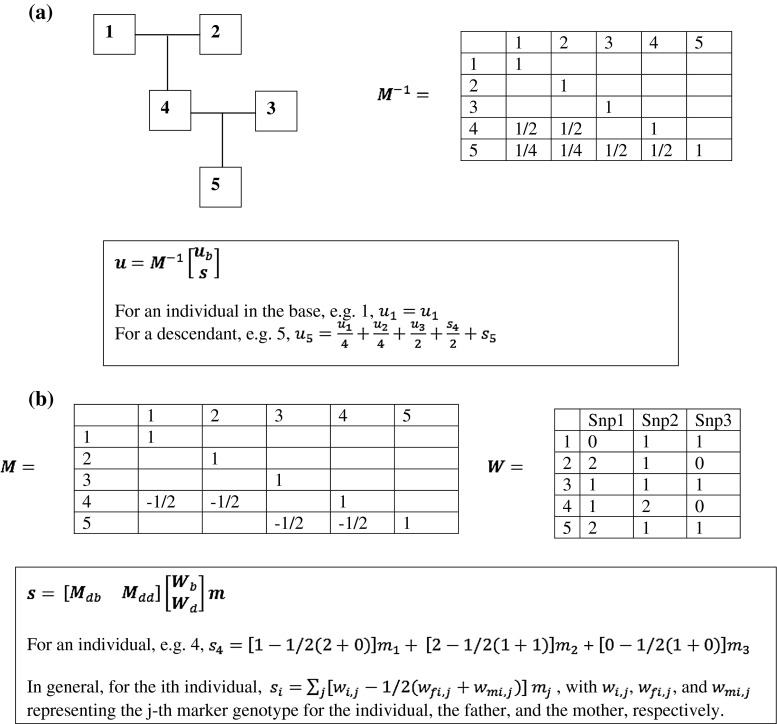
Genetic transmission and Mendelian sampling effects in the prediction model. **a** Transmission: genetic values of descendants are a function of genetic values of base individuals $\varvec{u}_{b}$ and Mendelian sampling effects predicted by ***s***. **b** Observed Mendelian sampling effects

Let **ϕ** be a vector of infinitesimal mendelian sampling effects which are deviations of individual genetic values from their respective parental averages. Then, the matrix operator **M** ^**−1**^ can be used to construct additive genetic values u as linear combinations of ancestor genetic values **u**_b_ and mendelian sampling **ϕ** of their descendants, as illustrated in part (a) of Fig. 1, so we can write:

$$\it \it {\mathbf{u}} = {\mathbf{M}}^{ - 1} \left[ {\begin{array}{*{20}c} {{\mathbf{u}}_{\text{b}} } \\ {\phi} \\ \end{array} } \right]$$

where **u** can be found by partitioning the **M** matrix in **M**_bb_ **, M**_dd_ **, M**_db_ and **M**_bd_ blocks, as:

$${\mathbf{M}} = \left[ {\begin{array}{*{20}c}    {{\mathbf{M}}_{{{\text{bb}}}} } \hfill & {{\mathbf{M}}_{{{\text{bd}}}} } \hfill  \\    {{\mathbf{M}}_{{{\text{db}}}} } \hfill & {{\mathbf{M}}_{{{\text{dd}}}} } \hfill  \\   \end{array} } \right],{\text{with}}\:{\mathbf{M}}_{{{\text{bb}}}}  = {\mathbf{I}},\,{\text{and}}\,{\mathbf{M}}_{{{\text{bd}}}}  = 0.$$

Using known results about the inverse of a lower triangular matrix, we obtain:

4 $${\mathbf{u}} = \left[ {\begin{array}{*{20}c} {\mathbf{I}} & {\mathbf{0}} \\ { - {\mathbf{M}}_{\text{dd}}^{ - 1} {\mathbf{M}}_{\text{db}} } & {{\mathbf{M}}_{\text{dd}}^{ - 1} } \\ \end{array} } \right]\left[ {\begin{array}{*{20}c} {{\mathbf{u}}_{\text{b}} } \\ {\varvec{\phi}} \\ \end{array} } \right] = \left[ {\begin{array}{*{20}c} {{\mathbf{u}}_{\text{b}} } \\ { - {\mathbf{M}}_{\text{dd}}^{ - 1} {\mathbf{M}}_{\text{db}} {\mathbf{u}}_{\text{b}} + {\mathbf{M}}_{\text{dd}}^{ - 1} {\varvec{\phi}} } \\ \end{array} } \right] = \left[ {\begin{array}{*{20}c} {{\mathbf{u}}_{\text{b}} } \\ {{\mathbf{u}}_{\text{d}} } \\ \end{array} } \right] $$

Equation (4) uses standard results under infinitesimal models developed when it was impossible to observe DNA, and a theoretical distribution was assigned to the unknown **ϕ** (see Quaas 1976). Availability of genotypes for progeny and parents gives a realized “molecular” mendelian sampling s, a predictor of** ϕ** which can be approached as a function of marked gene effects **m**:

5 $${\mathbf{s}} = \left[ {\begin{array}{*{20}c} {{\mathbf{M}}_{\text{db}} } & {{\mathbf{M}}_{{\text{dd}}} } \\ \end{array} } \right] \left[ {\begin{array}{*{20}c} {{\mathbf{W}}_{\text{b}} } \\ {{\mathbf{W}}_{\text{d}} } \\ \end{array} } \right]  {\mathbf{m}} $$

where matrices **W**_b_ and **W**_d_ contain the marker genotypes of base and descendant individuals, respectively. Figure 1b illustrates how expression (5) represents individual deviations from parental means, in terms of marked genetic effects, for a hypothetical genealogy of 5 individuals and 3 markers.

Then, replacing **ϕ** by s in (4), and using (5) in (4), with ${\mathbf{D}} = - {\mathbf{M}}_{\text{dd}}^{ - 1} {\mathbf{M}}_{\text{db}}$, we get:

6 $${\mathbf{u}}_{\text{d}} = {\mathbf{Du}}_{\text{b}} - {\mathbf{DW}}_{\text{b}} {\mathbf{m}} + {\mathbf{W}}_{\text{d}} {\mathbf{m}} = {\mathbf{Du}}_{\text{b}} + ({\mathbf{W}}_{\text{d}} - {\mathbf{DW}}_{\text{b}} ){\mathbf{m}} $$

And the model for phenotypes is then:

7 $${\mathbf{y}} = {\varvec{\mu}} + {\mathbf{Z}}_{\text{d}} {\mathbf{Du}}_{\text{b}} + {\mathbf{Z}}_{\text{d}}  (2{\mathbf{W}}_{\text{d}} - {\mathbf{DW}}_{\text{b}} ){\mathbf{m}} + {\mathbf{e}} $$

In the term **Z**_d_ **Du**_b_, **Z**_d_ (of order Ny × Nd) relates records to individuals (descendants d) and **D** relates individual genetic values to ancestors' genetic values **u**_b_ via simple coefficients of genome sharing (including consanguinity, i.e., multiple contributions of an ancestor to an individual). So this term in (7) concentrates all phenotype information of descendants to estimate the ancestors’ infinitesimal values. The term **Z**_d_ (2**W**_d_ − **DW**_b_) **m** in (7) groups two parts: **Z**_**d**_ **(W**_**d**_ − **DW**_**b**_ **) m**, the “molecular” mendelian sampling effects where individual marked effects deviate from ancestors’ marked effects, and **Z**_**d**_ **W**_**d**_ **m** which represents the direct relations between markers and phenotypes.

### Assumptions of the model

A set of possible assumptions is:

$$\begin{aligned}& {\mathbf{u}}_{{\mathbf{b}}} \sim {\mathbf{N}}\left( {{\mathbf{0}}, \, {\mathbf{I}}\varvec{\sigma}_{\varvec{u}}^{2} } \right) \hfill \\ &{\mathbf{m}}\sim {\mathbf{N}}\left( {{\mathbf{0}}, \, {\mathbf{I}}\varvec{\sigma}_{\varvec{m}}^{2} } \right) \hfill \\ &{\mathbf{Cov}}\left( {{\mathbf{u}}_{{\mathbf{b}}} ,{\mathbf{m}}} \right) = {\mathbf{0}} \hfill \\ \end{aligned}$$

The assumption of independent base individuals is usual in quantitative genetics. With DNA information and complete data it would be possible to make more general assumptions like ${\mathbf{u}}_{{\mathbf{b}}} \sim {\text{N}}\left( {\varvec{\mu}_{\varvec{u}} ,\;{\mathbf{H}}\sigma_{u}^{2} } \right),$ where **H** represents a genomic matrix, thus recognizing that individuals in the base populations may share genes. Again, the model is redundant if it is assumed that **u**_**b**_ = **W**_**b**_ **m** and ${\mathbf{H}} \, = {\mathbf{W}}_{{\mathbf{b}}} {\mathbf{W}}_{{\mathbf{b}}}^{'} \sigma_{m}^{2}$. Alternatively, model (7) can also accommodate fixed genetic values for individuals in the base population.

Distribution of marked effects **m** is assumed normal but other distributions such as the Gamma may be chosen, to take into account experimental results indicating few loci with large effects and many more loci with small effects (Goddard and Hayes 2009).

## Analyses of data

Firstly, repeated simulations were conducted to assess the predictive ability of the Mendelian segregation model MS (Eq. 7) relative to the marker model M (Eq. 2). Then, we analyzed a public sample simulated for the 12th European QTLMAS workshop by Lund et al. (2009), using several models including individual and marked genetic effects.

We preferred to use simulated data at this exploratory stage to understand the behavior of the compared models. Also, to simplify interpretation at this stage, estimation and prediction were limited to the unknowns in the models (**μ**, the vector of marked effects **m** and the vector of individual genetic values **u**) by applying known variances used to simulate the data.

We used the same statistical method BLUP to all models compared, which have either one (Eqs. 1 and 2) or two (Eqs. 3 and 7) random effects in addition to random residuals. BLUP of random effects were computed as detailed in the “Appendix”.

### Relative predictive performance of the Mendelian segregation (MS) model

Data were simulated using the QMSim software (Sargolzaei and Schenkel 2009). The simulated population had 1 base generation (25 individuals), 3 training generations (120 individuals) and the last generation (40 individuals) taken as prediction target. Mating was at random and the family size was 1. The simulated genome had 2 chromosomes of 1 Morgan each and 10 biallelic QTL/chromosome were responsible for the QTL fraction of genetic variance. Number of SNP markers used was either 2,000 or 200 per chromosome. Phenotypes in the base and target generations were simulated but not used to predict genetic values of the target generation. The phenotypes had variance 1 and overall heritability (infinitesimal + QTL effects) was 0.4. Three genetic scenarios were replicated 200 times: high (90 %), intermediate (50 %), or low (10 %) proportion of genetic variance explained by QTL.

**Fig. 2:**
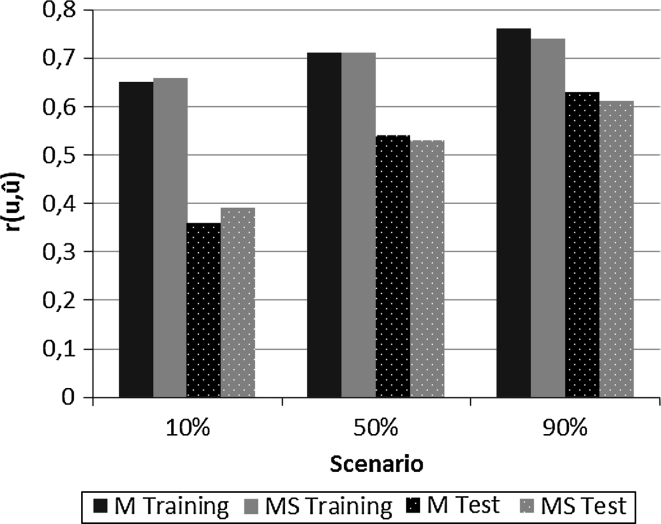
Accuracy of the marker (M) and Mendelian segregation (MS) models for the three simulation scenarios with 10, 50, or 90 % of the total genetic variance explained by QTL

Mean accuracies over 200 replicates when using 2,000 SNP markers are presented in Fig. 2 for 10, 50 and 90 % of total genetic variance explained by QTL. Accuracies were highest (0.76 for model M and 0.74 for model MS) in the training data when the genetic variance explained by QTL was high (90 %). The lowest correlations occurred for the test data under scenario 10 % (0.36 for M vs. 0.40 for MS). The MS model gave the best predictions when the infinitesimal effects were important (scenario 10 %) and model M gave the best predictions when QTL effects represented 90 % of genetic variance. Differences between mean accuracies of two models were small and non-significant (*P* < 0.05).

When fewer markers were used (200 SNP per chromosome), all accuracies were lower but the methods ranked as when using more (2,000 SNP per chromosome) markers (Table 1). The accuracy of the MS model was 12 % higher than that of the M model for the scenario with the 10 % of genetic variance explained by QTL and 5 % lower when the QTL explained the 90 % of total variance.

**Table 1:** Performance of the Mendelian segregation model: relative accuracies in the training and the test data

Simulated scenario	Training data (%)^a^	Test data(%)^a^
QTL variance 10 %		
200 SNP markers	103	112
2,000 SNP markers	102	108
QTL variance 50 %		
200 SNP markers	100	100
2,000 SNP markers	100	98
QTL variance 90 %		
200 SNP markers	99	95
2,000 SNP markers	97	97

^a^(%) is 100 times the ratio between the average accuracy under the Mendelian segregation model and the average accuracy under the marker model

### Analyses of a public simulated sample

In the data simulated for the 12th European QTLMAS workshop (Lund et al. 2009), the simulated phenotypes were influenced by 50 loci, including 15 major effect loci and 35 minor effect loci with a total heritability of 0.3. Marker information was available for 6,000 SNP (only 5,925 were polymorphic and used in our analyses) on 6 chromosomes. The population was simulated under random mating and the absence of selection. Each male was mated to 10 females and each mating pair produced 10 offspring. A data set of 4,665 individuals was split into a training set (3,165 individuals) and a test set (1,500). In the training set, the base population (generation 0) included 165 individuals with unknown parents. The remaining 3,000 individuals had known parents and were born in generations 1 and 2. The test set had 1,500 individuals born in generation 3 with complete genealogy. The targets of prediction were the simulated genetic values and phenotypes of the test individuals. The data used in prediction were the phenotypes of 3,000 individuals of generations 1 and 2, and the marker genotypes of all individuals.

Four models were compared using the known variances used for the simulation: the marker model (M) as in (2), the marker plus individual model (MI) as in (3), the marker plus mendelian effects model (MS) given in (7), and the individual model where the (co)-variance matrix of individual effects was the additive relationship **A** (individual infinitesimal model; II). The method to estimate the unknowns of all the models was BLUP. The known variances were given by Lund et al. (2009): $\sigma_{e}^{2} = 3.15 \,{\text{and}}\,  \sigma_{u}^{2} = 1.35$. The variance of marker effects was computed as $\sigma_{m}^{2} = \sigma_{u}^{2} /2\mathop \sum \nolimits_{j} p_{j} (1 - p_{j} )$. Correlations between predicted values and simulated genetic values and phenotypes for the training and test populations are given in Table 2. The goodness of fit of model (7) for the training data was moderate $r\left( {\left( {\widehat{{\mathbf{u}}},{\mathbf{y}}} \right) = 0.53} \right)$ but it yielded the best predictions for genetic values $r\left( {\left( {\widehat{{\mathbf{u}}},{\mathbf{u}}} \right) = 0.94} \right)$ and phenotypes $r\left( {\widehat{{\mathbf{u}}},{\mathbf{y}}} \right) = 0.55$ in the test sample. Model [7] was also the best to estimate the marked effects **m**: the correlations between estimates of **m** and the simulated allele substitution effects, in absolute values, were 0.69 for Model [7] and 0.56 for both the marker model and the “marker + individual” model.

**Table 2:** Correlations between the predicted genetic values $\left( {\widehat{{\mathbf{u}}}} \right)$, simulated genetic values (**u**), and simulated phenotypes (***y***) in the training and test data

Model^a^	M	MI	MS	II
Training data				
${\text{r}}(\widehat{{\mathbf{u}}},{\mathbf{u}})$	0.87	0.84	0.94	0.69
${\text{r}}(\widehat{{\mathbf{u}}},{\mathbf{y}})$	0.59	0.77	0.53	0.74
Test data				
${\text{r}}(\widehat{{\mathbf{u}}},{\mathbf{u}})$	0.81	0.77	0.94	0.43
${\text{r}}(\widehat{{\mathbf{u}}},{\mathbf{y}})$	0.46	0.46	0.55	0.27

^a^Models. M: marker model (Eq. 2); MI: marker plus individual effect model (Eq. 3); MS: Mendelian segregation model (Eq. 7); II: individual infinitesimal model based on pedigree (Eq. 1)

## Discussion

As reviewed in the Introduction, there are plausible arguments to combine marked effects models with other individual effects when analyzing complex traits. To do so, the strategy used in the MS model [7] is to decompose the individual genetic value into two terms: a contribution from base individuals, weighted by the transmission matrix **D**, and a contribution from mendelian sampling occurring at several meiosis from base individuals to their descendants, instead of attempting to fit twice the additive genetic value of an individual as in model [3]. In traditional infinitesimal models, mendelian sampling is an unknown theoretical random term, so predictions of future phenotypes (of future progeny) are based on ancestor phenotypes and random terms. At present, with the availability of numerous markers, mendelian sampling is realized for each individual and it can be used to improve predictions.

Model [7] builds on very well-known results in quantitative genetics. Early work described how genetic transmission operates in the additive relationship matrix **A** (e.g., Quaas 1976 and Henderson 1976, who presented detailed factorizations of the **A** matrix). Subsequent models included genetic transmission at unobserved segregating QTL (e.g., Fernando and Grossman 1989; Meuwissen and Goddard 2000; Legarra and Fernando 2009) and combined within family and between family marker effects in the context of methodology for QTL search (e.g., Abecasis et al. 2000). In animal breeding, efforts have focused on combining genotype data with genealogy data in individual genomic models, as reviewed by Meuwissen et al. (2011). The model [7] developed here builds on previous work by the simultaneous inclusion of infinitesimal and marked genetic effects. In this way the model might capitalize on two advantages of molecular information: the improvement of the infinitesimal prediction by the estimation of realized mendelian sampling in descendant individuals, and by capturing marked gene effects without bias due to family structure, i.e., to predict marked effects and infinitesimal effects simultaneously and without redundancy. Here, marked effects are estimated at the level of the population (marked effects **m** in model MS [7] are not defined within family) but the family structure is taken into account in the estimation model.

Results of simulations indicate that the predictive ability of the MS model is comparable to that of the marker model. On one hand, the accuracies obtained in different genetic scenarios suggest that the MS model might be useful when markers are not adequate to fully explain the genetic background (low QTL variances with high infinitesimal variance, or low marker density).

On the other hand, the marker model M yielded slightly higher predictive ability than MS when QTL were important and marker density was high. This result might reflect sub-optimality of the MS model to exploit favorable situations where markers do effectively capture much of total genetic variance. This might be explained by the simple distributional assumptions that we assumed at this exploratory stage for the base individuals and the marked effects of model MS in [7] and accompanying assumptions. In particular, the marker model [2], and, more explicitly, its equivalent model “Genomic BLUP”, capitalizes the complete data setting studied here by estimating covariances among base individuals, and covariances between base individuals and descendants. So, for the MS model to be fully competitive, its distributional assumptions should be extended to take into account those relationships.

Results for the QTLMAS example are encouraging but unique and different from those of replicated simulations. At least two reasons may be advanced to explain these different results: the more complicated genetic background and the large family size, a full-sib design, simulated in the QTLMAS data set. But the impact of such factors on predictive ability needs further investigation.

Further investigation is also needed on variance component estimation of models including marker and individual effects. Duchemin et al. (2012) were able to estimate both components of variance from real data using model [3], i.e., the variance of individual effects and the variance of marker effects. We are currently studying variance components estimation for model [7], with infinitesimal effects defined only for the base individuals and variance structure designed to avoid identifiability problems.

Also, at this stage of model development, we are assuming complete data, in particular genotypes of base individuals. In some situations, it may possible to impute missing data. Also, if genealogy is unknown and if all individuals are in the genotyped sample, parent-progeny pairs can be easily identified using DNA data (Rohlfs et al. 2012). However, to cover many variable situations in real life, it should be necessary to expand model [7] to include heterogeneous variances where mendelian sampling is observed for some individuals but it remains a random value for individuals without genotyped parents.

Another potential improvement of the MS model in [7] is the representation of genetic transmission (as in expression [5]) and marked genetic effects (as in [2] and [7]) which may be certainly improved. Haplotypes can be used instead of single non-phased SNP. The model is also compatible with approaches where some QTL are known, markers are preselected or markers are weighted by their effects during prediction (e.g. Zhang et al. 2011).

## Conclusions

According to the literature on prediction of complex traits, it is justified to keep, both, individual (infinitesimal) and marked gene effects in the statistical predictive model. We gave a formal derivation of a mendelian sampling MS model where individual effects are a function of infinitesimal effects of base individuals and mendelian sampling in descendants, traced using available DNA data. At this stage of research, we are assuming complete data, simple distributional assumptions for individual and marked genetic effects, and known variances. First simulation results suggest that these simplifying assumptions should be extended to render the MS model fully competitive.

## Appendix: Computation of individual and marked genetic effects using BLUP

Let ${{\upsigma}}_{\text{i}}^{2}$, ${{\upsigma}}_{\text{M}}^{2}$, and ${{\upsigma}}_{\text{e}}^{2}$ be the variance of infinitesimal effects, the genetic variance due to all QTL, and the residual variance, respectively. Also the variance of individual markers is ${{\upsigma}}_{\text{m}}^{2} = {{\upsigma}}_{\text{M}}^{2} /{\text{k}}$, with ${\rm{k}} = 2\sum\nolimits_{{\rm{j}}} {{\rm{p}}_{{\rm{j}}} \left( {1 - {\rm{p}}_{{\rm{j}}} } \right)}$. Then:

$$\begin{aligned} \alpha_{u} &= {{\upsigma}}_{\text{e}}^{2} / \left( {{{\upsigma}}_{\text{i}}^{2} + {{\upsigma}}_{\text{M}}^{2} } \right) \hfill \\ \alpha_{m} &= {{\upsigma}}_{\text{e}}^{2} / {{\upsigma}}_{\text{m}}^{2} \hfill \\ \alpha_{i} &= {{\upsigma}}_{\text{e}}^{2} / {{\upsigma}}_{\text{i}}^{2} \hfill \\ \end{aligned}$$

Solutions for models compared were:

For model [1]:

$$\left[ {\begin{array}{*{20}c} {\widehat{{{\upmu}}}} \\ {\widehat{{\mathbf{u}}}} \\ \end{array} } \right] = \left[ {\begin{array}{*{20}c} {1^{ '} 1} & {1^{ '} {\mathbf{Z}}} \\ {{\mathbf{Z}}^{ '} 1} & {{\mathbf{Z}}^{ '} {\mathbf{Z}} + {{\upalpha}}_{\text{u}} {\mathbf{A}}^{ - 1} } \\ \end{array} } \right]^{ - 1} \left[ {\begin{array}{*{20}c} {1^{ '} {\mathbf{y}}} \\ {{\mathbf{Z}}^{ '} {\mathbf{y}}} \\ \end{array} } \right]$$

where 1 is a vector of 1 and **Z** is the incidence matrix. $\widehat{{{\upmu}}}$ is the BLUE (best linear unbiased estimator) of the general mean, and $\widehat{{\mathbf{u}}}$ is the solution for individual effects.

For model [2]:

$$\left[ {\begin{array}{*{20}c} {\widehat{{{\upmu}}}} \\ {\widehat{{\mathbf{m}}}} \\ \end{array} } \right] = \left[ {\begin{array}{*{20}c} {1^{ '} 1} & {1^{ '} {\mathbf{X}}} \\ {{\mathbf{X}}^{ '} 1} & {{\mathbf{X}}^{ '} {\mathbf{X}} + {{\upalpha}}_{\text{m}} {\mathbf{I}}^{{}} } \\ \end{array} } \right]^{ - 1} \left[ {\begin{array}{*{20}c} {1^{ '} {\mathbf{y}}} \\ {{\mathbf{X}}^{ '} {\mathbf{y}}} \\ \end{array} } \right]$$

where X = ***ZW***, i.e., the incidence matrix times the matrix of genotypes, centered by column. $\widehat{{\mathbf{m}}}$ is the solution for marked effects.

Predictions from model [2] can be also obtained with the individual model:

$$\left[ {\begin{array}{*{20}c} {\widehat{{{\upmu}}}} \\ {\widehat{{\mathbf{u}}}} \\ \end{array} } \right] = \left[ {\begin{array}{*{20}c} {1^{ '} 1} & {1^{ '} {\mathbf{Z}}} \\ {{\mathbf{Z}}^{ '} 1} & {{\mathbf{Z}}^{ '} {\mathbf{Z}} + {{\upalpha}}_{\text{u}} {\mathbf{G}}^{ - 1} } \\ \end{array} } \right]^{ - 1} \left[ {\begin{array}{*{20}c} {1^{ '} {\mathbf{y}}} \\ {{\mathbf{Z}}^{ '} {\mathbf{y}}} \\ \end{array} } \right]$$

where $${\mathbf{G}} = {\mathbf{WW}}^{ '} /{\text{k}}$$

For model [3]:

$$\left[ {\begin{array}{*{20}c} {\widehat{{{\upmu}}}} \\ {\widehat{{\mathbf{u}}}} \\ {\widehat{{\mathbf{m}}}} \\ \end{array} } \right] = \left[ {\begin{array}{*{20}c} {1^{ '} 1} & {1^{ '} {\mathbf{X}}_{1} } & {1^{ '} {\mathbf{X}}_{2} } \\ {{\mathbf{X}}_{1}^{ '}  1} & {{\mathbf{X}}_{1}^{ '} {\mathbf{X}}_{1} + {{\upalpha}}_{\text{u}}  {\mathbf{A}}^{ - 1} } & {{\mathbf{X}}_{1}^{ '} {\mathbf{X}}_{2} } \\ {{\mathbf{X}}_{2}^{ '} 1} & {{\mathbf{X}}_{2}^{ '} {\mathbf{X}}_{1} } & {{\mathbf{X}}_{2}^{ '} {\mathbf{X}}_{2} + {{\upalpha}}_{\text{m}} {\mathbf{I}}} \\ \end{array} } \right]^{ - 1} \left[ {\begin{array}{*{20}c} {1^{ '} {\mathbf{y}}} \\ {{\mathbf{X}}_{1}^{ '} {\mathbf{y}}} \\ {{\mathbf{X}}_{2}^{ '} {\mathbf{y}}} \\ \end{array} } \right]$$

where ${\text{X}}_{1} = {\text{Z}}$ and ${\text{X}}_{2} = {\text{ZW}}$. $\widehat{{\mathbf{u}}}$ is the solution for individual effects and $\widehat{{\mathbf{m}}}$ is the solution for marked effects.

For model [7]:

$$\left[ {\begin{array}{*{20}c} {\widehat{{{\upmu}}}} \\ {\widehat{{{\mathbf{u}}_{\text{b}} }}} \\ {\widehat{{\mathbf{m}}}} \\ \end{array} } \right] = \left[ {\begin{array}{*{20}c} {1^{ '} 1} & {1^{ '} {\mathbf{X}}_{1} } & {1^{ '} {\mathbf{X}}_{2} } \\ {{\mathbf{X}}_{1}^{ '} 1} & {{\mathbf{X}}_{1}^{ '} {\mathbf{X}}_{1} + {{\upalpha}}_{\text{i}} {\mathbf{I}}} & {{\mathbf{X}}_{1}^{ '} {\mathbf{X}}_{2} } \\ {{\mathbf{X}}_{2}^{ '} 1} & {{\mathbf{X}}_{2}^{ '} {\mathbf{X}}_{1} } & {{\mathbf{X}}_{2}^{ '} {\mathbf{X}}_{2} + {{\upalpha}}_{\text{m}} {\mathbf{I}}} \\ \end{array} } \right]^{ - 1} \left[ {\begin{array}{*{20}c} {1^{ '} {\mathbf{y}}} \\ {{\mathbf{X}}_{1}^{ '} {\mathbf{y}}} \\ {{\mathbf{X}}_{2}^{ '} {\mathbf{y}}} \\ \end{array} } \right]$$

where ${\mathbf{X}}_{1} = {\mathbf{Z}}_{\text{d}} {\mathbf{D}}\;{\text{and}}\;{\mathbf{X}}_{2} = {\mathbf{Z}}_{\text{d}} 2\left( {{\mathbf{W}}_{\text{d}} - {\mathbf{DW}}_{\text{b}} } \right),$ and $\widehat{{u_{b} }}$ is the solution for base individuals and $\widehat{{\mathbf{m}}}$ is the solution for marked effects.

## References

[CR1] Abecasis GR, Cardon RL, Cookson WO (2000). A general test of association for quantitative traits in nuclear families. Am J Hum Genet.

[CR2] Aguilar I, Misztal I, Legarra A, Tsuruta S (2011). Efficient computation of the genomic relationship matrix and other matrices used in single-step evaluation. J Animal Breed Genet.

[CR3] De Los campos G, Naya H, Gianola D, Crossa J, Legarra A (2009). Predicting quantitative traits with regression models for dense molecular markers and pedigree. Genetics.

[CR4] Duchemin SI, Colombani C, Legarra A, Baloche G, Larroque H (2012). Genomic selection in the French Lacaune dairy sheep breed. J Dairy Sci.

[CR5] Fernando RL, Grossman M (1989). Marker assisted selection using best linear unbiased prediction. Genet Sel Evol.

[CR6] Gianola D, De Los Campos G, Hill WG, Manfredi E, Fernando R (2009). Additive genetic variability and the Bayesian alphabet. Genetics.

[CR7] Goddard ME (2009). Genomic selection: prediction of accuracy and maximisation of long term response. Genetica.

[CR8] Goddard ME, Hayes BJ (2009). Mapping genes for complex traits in domestic animals and their use in breeding programmes. Nat Rev Genet.

[CR9] Hazel LN (1943). The genetic basis for constructing selection indexes. Genetics.

[CR10] Henderson CR (1975). Best linear unbiased estimation and prediction under a selection model. Biometrics.

[CR11] Henderson CR (1976). Simple method for computing inverse of a numerator relationship matrix used in prediction of breeding values. Biometrics.

[CR12] Legarra A, Fernando RL (2009). Linear models for joint association and linkage QTL mapping. Genet Sel Evol.

[CR13] Lund MS, Sahana G, De Koning DJ, Su G, Carlborg O (2009). Comparison of analyses of the QTLMAS XII common dataset. I: genomic selection. BMC Proc.

[CR14] Meuwissen TH, Goddard ME (2000). Fine mapping of quantitative trait loci using linkage disequilibria with closely linked marker loci. Genetics.

[CR15] Meuwissen TH, Hayes BJ, Goddard ME (2001). Prediction of total genetic value using genome-wide dense marker maps. Genetics.

[CR16] Meuwissen TH, Luan T, Woolliams JA (2011). The unified approach to the use of genomic and pedigree information in genomic evaluations revisited. J Anim Breed Genet.

[CR17] Quaas RL (1976). Computing diagonal elements and inverse of a large numerator relationship matrix. Biometrics.

[CR18] Rohlfs RV, Fullerton SM, Weir BS (2012). Familial identification: population structure and relationship distinguishability. PLoS Genet.

[CR19] Sargolzaei M, Schenkel FS (2009) QMSim: a large-scale genome simulator for livestock. Bioinformatics 25:680–681. First published January 28, 2009, doi:10.1093/bioinformatics/btp045 10.1093/bioinformatics/btp045 19176551

[CR20] Vanraden P (2008). Efficient method to compute genomic predictions. J Dairy Sci.

[CR21] Yang JT, Manolio A, Pasquale LR, Boerwinkle E, Caporaso N (2011). Genome partitioning of genetic variation for complex traits using common SNPs. Nat Genet.

[CR22] Zhang Z, Ding X, Liu J, De Koning DJ, Zhang Q (2011). Genomic selection for QTL-MAS data using a trait-specific relationship matrix. BMC Proc.

